# Novel device for creating continuous curvilinear capsulorhexis

**DOI:** 10.1186/s40064-016-3736-6

**Published:** 2016-12-01

**Authors:** Mustafa Soylak

**Affiliations:** Mechatronics Laboratory, Faculty of Aeronautics and Astronautics, Erciyes University, 38039 Kayseri, Turkey

**Keywords:** Capsulorhexis, Novel design, Cataract surgery, IOL

## Abstract

**Purpose:**

The purpose of this paper is to develop a novel capsulorhexis system.

**Setting:**

Mechatronics Laboratory, University of Erciyes and Kayseri Maya Eye Hospital.

**Design:**

A 3D model was created and simulations were conducted to develop a new device which was designed, fabricated and tested for continuous curvilinear capsulorhexis (CCC). The name of this system is the electro-mechanical capsulorhexis system (EMCS).

**Methods:**

The 3D model was created by using a commercial design software and a 3D printer was used to fabricate the EMCS Finite element analysis and geometrical relation tests of the EMCS for different sized lenses were performed.

**Results:**

The results show that the EMCS is a perfect solution for capsulorhexis surgeries, without mechanical or geometrical problems.

**Conclusions:**

The EMCS can open the anterior lens capsule more easily and effectively than manual CCC applications and needs less experience.

## Background

According to the World Health Organization (WHO), cataract, which is one of the most important reasons for sight loss, is an eye disease and it causes the natural lens within the eye to lose transparency. Cataract may be caused by trauma to the eye, diabetes, eye infection or using certain medicines. However, the most important reason for cataract is old age. The only treatment for cataract is to perform a surgical operation; it cannot be cured via medicine. During this surgical operation, the natural lens which has lost its transparency is replaced with an artificial lens (i.e. intra ocular lens, IOL). In the past, lens removal through a large limbal incision was the most common surgical application for cataract (Abellán et al. 2013).

This procedure is called intracapsular cataract extraction (ICCE). Patients experienced many surgical complications after this surgical operation. Because of these complications, surgeons reapplied extracapsular cataract extraction (ECCE). This technique was based on creating an opening, known as a “capsulotomy”, in the anterior capsular bag through which the lens nucleus could be “prolapsed” (Mohammadpour et al. 2012). During this operation, using a small cross-section of about 1.5 mm, the inside of the eye is reached and a small piece is extracted in order to remove the cataractous lens from the anterior capsule. Then, via ultrasonic waves, the functionless lens is destroyed and removed via vacuum. Finally, an artificial lens is placed in the same region. The most important step is to make a circular incision in the anterior capsule to remove the cataractous lens. This step is called capsulorhexis. The importance of consistently achieving a symmetrically round and properly sized capsulorhexis has increased because the surgeon’s main target is to fit newer refractive IOLs. In order to maximize the performance of the capsulorhexis step, several approaches have been developed (Gimbel and Neuhann 1990, 1991; Pande 1993; Gimbel 2007).

In addition, the development and improvement of devices and the use of more adhesives in surgical procedures have resulted in significant improvements in the technique and in the reduction of post surgery complications. At present, cataract surgery is carried out using a microincision and this involves fewer risks for the patients (Gimbel and Neuhann 1991).

Currently, in operations which are conducted using the manual capsulorhexis method, special devices such as the ring calliper (the circular ring that indicates the borders of the capsulorhexis) (Tassignon et al. 2006; Dhubhghaill et al. 2015) and special forceps are employed. In addition, femto laser applications in cataract operations are commonly being applied (Donaldson et al. 2013; Grewal et al. 2016; Ranka and Donnenfeld 2015). In capsulorhexis applications which are performed using laser, surgical procedures such as making a window on the wall of the capsule and breaking up the damaged lens and preparing it for phacoemulsification are conducted with high precision and minimum error. However, as the laser devices are very expensive they can not be used extensively.

Recently, new nonfemtosecond-laser technologies have been introduced such as the CapsuLaser (thermal laser) (Stodulka 2015; Packard 2015), Zepto capsulorhexis device and ApetureRx (Kontos 2015).

The best and most approved method for capsulorhexis treatment is continuous curvilinear capsulorhexis (CCC). Capsulorhexis has two different procedures: Anterior Continuous Curvilinear Capsulorhexis (ACCC) and Posterior Continuous Curvilinear Capsulorhexis (PCCC). The critical step for both capsulorhexis applications is the making of a window in the capsule wall. In this study the author developed a device for anterior capsulorhexis alone. The system developed is not intended for use in posterior capsulorhexis. Before the EMCS, trypan blue, which is a special dye to increase the visibility of the capsule area, was widely used in capsulorhexis applications. With the help of the device developed in this study, there is no need to use trypen blue. However, as in current studies, the anterior chamber must be filled with cohesive viscoelastic in EMCS applications.

In this method by obtaining the correct dimensions for capsulorhexis application, surgeons enhance surgical safety, hydrodissection, cortical cleanup, circularity and good centration of the intra ocular lens (IOL). The most important disadvantage of this method is that it completely depends on the surgeon’s expertise. Moreover, the fact that it requires time to train surgeons in the technique and the possibility of negative results during this period are its other disadvantages. In this study, a new device for CCC, which requires minimal surgical experience, was developed. The design, prototype and simulation studies are presented.

## Description of system

A new type of instrument to optimize the time, size, shape, and centration of the capsulorhexis during intra ocular lens (IOL) surgery was designed and fabricated. This tool is positioned and fixed on the eye’s surface by using a vacuum component. When it is in position, it provides an ideal guide for the surgeon to follow and facilitate optimal capsulorhexis shape and centration. This system is called the EMCS (electro-mechanical capsulorhexis system). The EMCS has an end effector mechanism to make the CCC Three cutting methods can be applied in the EMCS by using different end effectors.

These methods include use of the following:

1. Heated wire
2. Laser probe
3. Metal knife

**Fig. 1:**
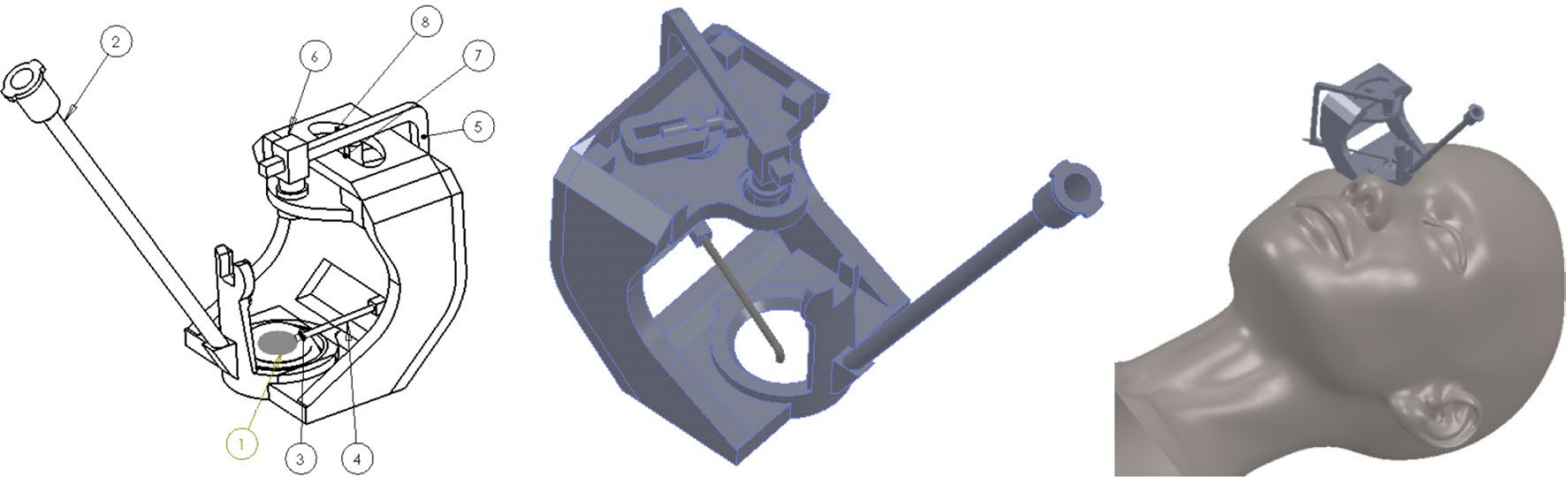
EMCS and components

The EMCS’s components are as shown Fig. 1:

1. Eye and cutting edge (5–6 mm diameter size)
2. Vacuum component
3. Incision area
4. Cutting tool
5. Cutting tool holder
6. Sliding guide (1)
7. Rotating electrical motor
8. Sliding Guide (2)

**Fig. 2:**
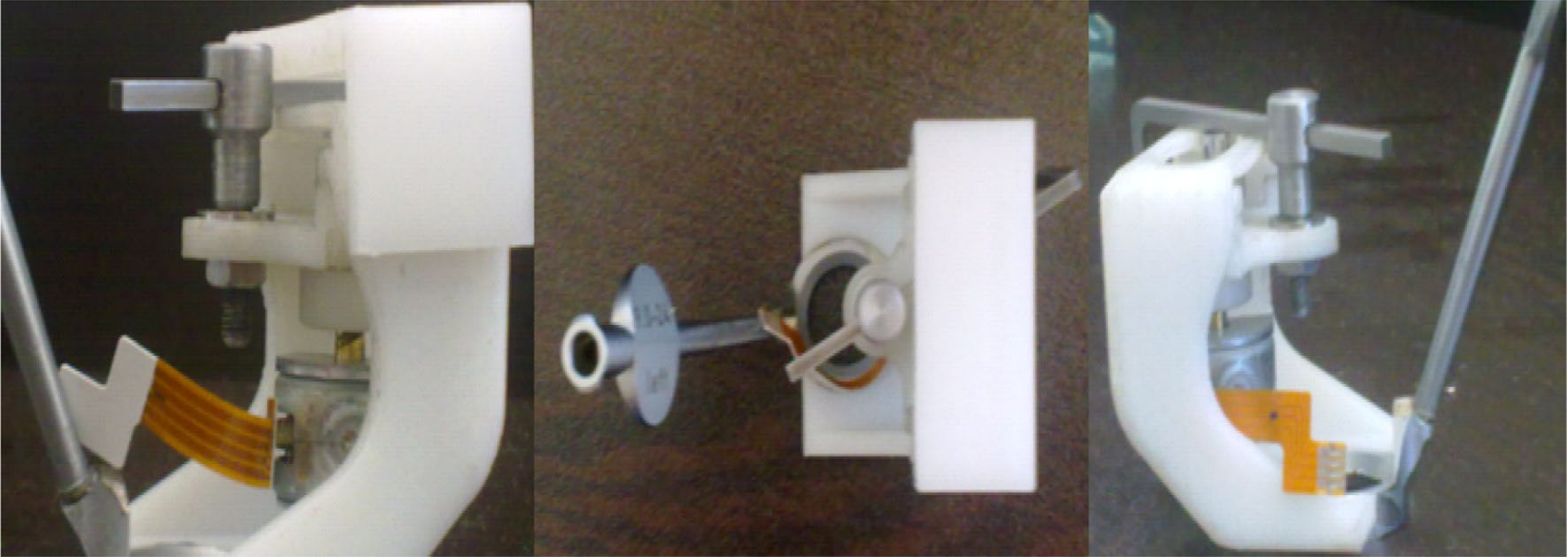
Experimental model of the EMCS

An experimental model of the EMCS was built using a 3D printer. This model is shown in Fig. 2.

The EMCS is 4 cm wide, 5 cm long and 6 cm in height. There is a vacuum component (2) to hold the eye (1) and this provides support for surgeons to avoid any movement or vibration of the eye. At the same time, this component carries all of the other system parts. The vacuum component (2) must be changed before surgery to make the capsulorhexis application suitable for patient data and to make the device behave according to the variable diameter of the corneal curvature. If any movement or vibration occurs, all of the system’s parts make the same movement owing to the system’s design. The cutting tool (4) enters the eye by means of an incision (3) which is made by the surgeon. The size of this incision is 1.5 mm.

After this, the electrical motor (7) starts to work and creates a circular movement on the motor’s shaft. The sliding guide (2) is a very important component in this study. The shape of this guide has a relation with the circular shape of the cutting tool (4). The cutting tool makes an adjustable circular movement with the cutting tool holder following the sliding guide (2). The sliding guide (1) gives linear movement freedom to the cutting tool holder when it follows the sliding guide (2). Identification of this relation and the EMCS parts were designed by Solidworks^®^ commercial design software.

The following parameters were analyzed using a simulation of the EMCS: diameter, centration, and regularity of the capsulorhexis, and duration of the rhexis procedure, as well as intraoperative complications (capsular tear, posterior capsule rupture). The simulation results showed that the EMCS can perform CCC without any deviation.

## Simulation results and discussions

### FEM result

To simulate the mechanical behavior of the proposed CCC system when the power applied by the DC motor is on the motor shaft, the finite element method (FEM) is applied. This section presents a static analysis of the behavior of the CCC under power from the DC motor shaft, and shows the maps of the results of the structural stresses. To apply this, the geometric modeling of the CCC in 3D was made on the Solidworks^®^ commercial program. After that, the physical properties of the EMCS were modeled.

**Fig. 3:**
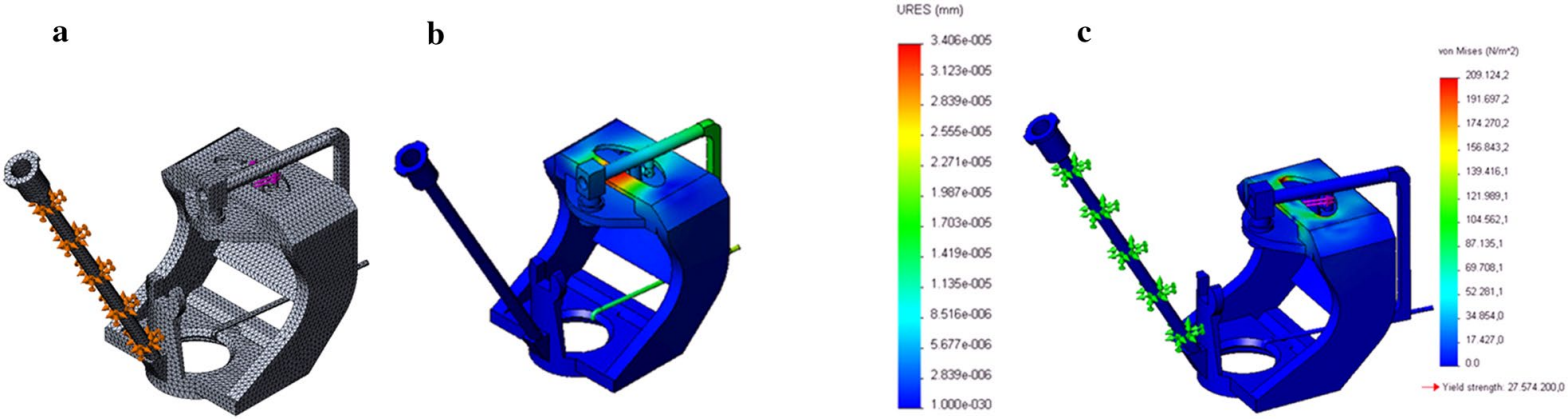
**a** Meshing of the EMCS Surface. **b** Displacement map of the EMCS. **c** Nonlinear nodal stress analysis of the EMCS (provided by Solidworks^®^)

 Later, the boundary conditions and loads were placed in order to perform the meshing of the EMCS (see Fig. 3). The resulting graph of the stresses and elastic deformation was obtained. The physical properties of the EMCS (i.e. the materials with which the components were made) are shown in Table 1. These parameters are required by Solidworks^®^ to perform the analysis.

**Table 1:** EMCS parameters

Property	Value	Units
Elastic modulus	6.9 e+010	N/m^2^
Poissons ratio	0.33	N/A
Shear modulus	2.7 e+010	N/m^2^
Density	2700	kg/m^3^
Tensile strength	68,935,600	N/m^2^
Yield strength	27,574,200	N/m^2^
Thermal expansion coefficient	2.4 e−005	/K
Thermal conductivity	200	W/(m K)
Specific heat	900	J/(kg K)

### Result of the geometric relation analysis

**Fig. 4:**
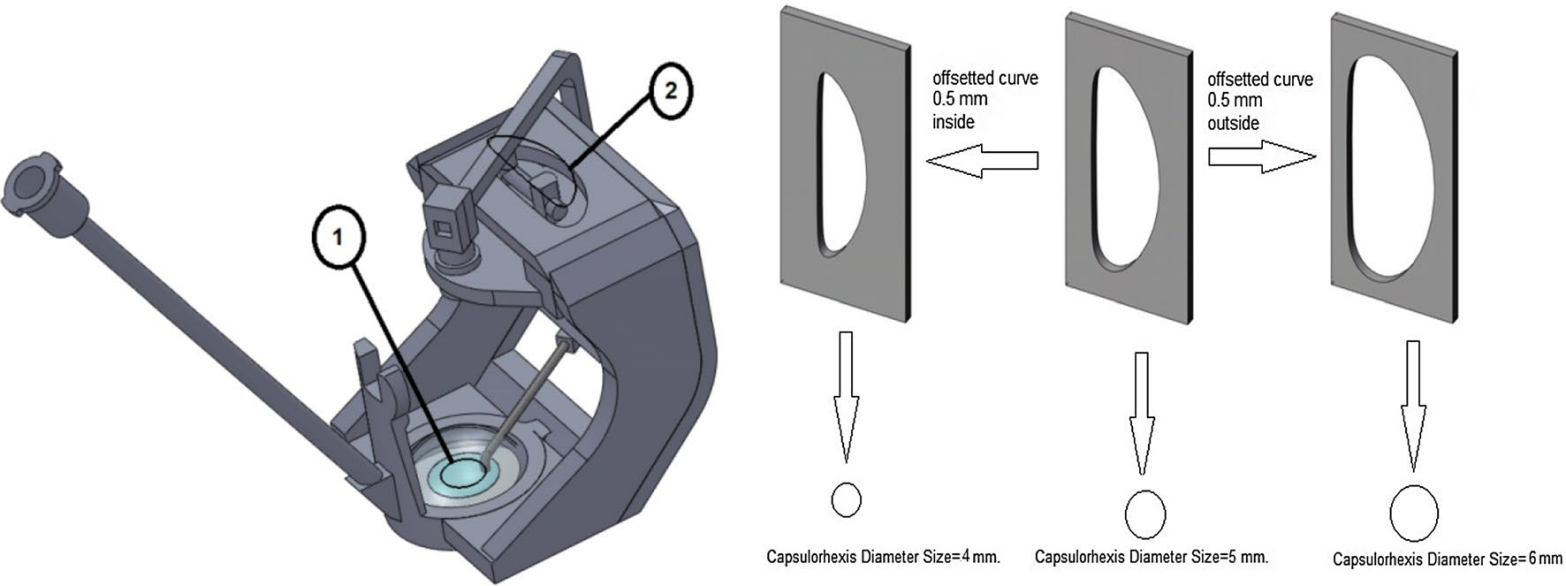
Relation between size of the guide curve and capsulorhexis diameter

The system developed in this study follows the guide curve (2) which is a mechanical aquaplaning element and is activated via the DC motor. Following this, a circular capsulorhexis movement is obtained at the tip point. Simulation studies show that by using different guide curves of different dimensions, various capsulorhexis diameters are obtained. Therefore, this system can be applied on patients with eye lenses of different diameters (see Fig. 4).

## Conclusions

In this study, a novel system is developed for capsulorhexis applications. The system developed eliminates the time needed to train surgeons, which is one of the most important problems in operations for capsulorhexis application. Moreover, it is possible to use different cutting techniques. It is called the electro-mechanical capsulorhexis system (EMCS). The EMCS can be used on different sized lenses by making small mechanical changes. When we compare the EMCS with other capsulorhexis systems, a laser probe, heated wire or metal knife can be chosen as the cutting tool, thus the operator of the EMCS can benefit from all the advantages of the selected cutting tool. The EMCS is more economical than all the other systems except for manual capsulorhexis. It is more hygenic because of its changeable cutting tool. The operator is required to do less tasks during the operation compared with other systems. In the circular cutting process performed by the EMCS, the cutting speed and surface quality are higher owing to the continuous and smooth movements. However, the EMCS has two main disadvantages; one of them is its heavy body and the other is its complex structure. The finite element method (FEM) and geometrical relation analysis were applied to the EMCS to show the mechanic behavior of the system. Analysis of the EMCS showed that the most common capsulorhexis technique of CCC can be applied on patients more easily, more economically and more reliably by using the EMCS
